# Schistosomes Induce Regulatory Features in Human and Mouse CD1d^hi^ B Cells: Inhibition of Allergic Inflammation by IL-10 and Regulatory T Cells

**DOI:** 10.1371/journal.pone.0030883

**Published:** 2012-02-08

**Authors:** Luciën E. P. M. van der Vlugt, Lucja A. Labuda, Arifa Ozir-Fazalalikhan, Ellen Lievers, Anouk K. Gloudemans, Kit-Yeng Liu, Tom A. Barr, Tim Sparwasser, Louis Boon, Ulysse Ateba Ngoa, Eliane Ngoune Feugap, Ayola A. Adegnika, Peter G. Kremsner, David Gray, Maria Yazdanbakhsh, Hermelijn H. Smits

**Affiliations:** 1 Department of Parasitology, Leiden University Medical Center, Leiden, The Netherlands; 2 Department of Pulmonology, Erasmus Medical Center, Rotterdam, The Netherlands; 3 Institute for Immunology and Infection Research, School of Biological Sciences, University of Edinburgh, Edinburgh, United Kingdom; 4 Institute of Infection Immunology, Twincore/Centre for Experimental and Clinical Infection Research, Hanover, Germany; 5 Department of Cell Biology, Bioceros B.V., Utrecht, The Netherlands; 6 Medical Research Unit, Albert Schweitzer Hospital, Lambaréné, Gabon; 7 Institute of Tropical Medicine, University of Tübingen, Tübingen, Germany; Fundação Oswaldo Cruz, Brazil

## Abstract

Chronic helminth infections, such as schistosomes, are negatively associated with allergic disorders. Here, using B cell IL-10-deficient mice, *Schistosoma mansoni*-mediated protection against experimental ovalbumin-induced allergic airway inflammation (AAI) was shown to be specifically dependent on IL-10-producing B cells. To study the organs involved, we transferred B cells from lungs, mesenteric lymph nodes or spleen of OVA-infected mice to recipient OVA-sensitized mice, and showed that both lung and splenic B cells reduced AAI, but only splenic B cells in an IL-10-dependent manner. Although splenic B cell protection was accompanied by elevated levels of pulmonary FoxP3^+^ regulatory T cells, *in vivo* ablation of FoxP3^+^ T cells only moderately restored AAI, indicating an important role for the direct suppressory effect of regulatory B cells. Splenic marginal zone CD1d^+^ B cells proved to be the responsible splenic B cell subset as they produced high levels of IL-10 and induced FoxP3^+^ T cells *in vitro*. Indeed, transfer of CD1d^+^ MZ-depleted splenic B cells from infected mice restored AAI. Markedly, we found a similarly elevated population of CD1d^hi^ B cells in peripheral blood of *Schistosoma haematobium*-infected Gabonese children compared to uninfected children and these cells produced elevated levels of IL-10. Importantly, the number of IL-10-producing CD1d^hi^ B cells was reduced after anti-schistosome treatment. This study points out that in both mice and men schistosomes have the capacity to drive the development of IL-10-producing regulatory CD1d^hi^ B cells and furthermore, these are instrumental in reducing experimental allergic inflammation in mice.

## Introduction

The prevalence and severity of allergic diseases and asthma has increased over the last five decades in industrialized countries [1]. Conversely, lower prevalence of allergic diseases is seen in low income countries. Many epidemiological studies have reported an inverse association between helminth infections, which are highly prevalent in developing countries, and allergic or auto-immune disorders [2]–[4]. In order to study the interaction between helminth infections and protection against allergic diseases, murine models of allergic airway inflammation (AAI) and helminth infection have been developed. For example, *H. polygyrus* or *T. spiralis* infections protected against house dust mite-induced and/or ovalbumin (OVA)-specific AAI [5], [6]. In addition, *S. mansoni* infection protected mice against OVA-induced airway hyperresponsiveness (AHR)[7]; protection was optimal during the chronic stage of infection, but not the acute stage [8].

Helminth infections are characterized by potent type 2 effector responses and a strong regulatory network [9]. Regulatory T (Treg) cells are well-known for their suppressive capacity, but recent studies in auto-immunity models have indicated that also B cells can be important players in immune regulation [10]. These so-called regulatory B cells are known to influence the immune system by the regulatory cytokines IL-10 and TGF-β, influencing T cell proliferation, downregulating CD4^+^, CD8^+^, NK T cell activation and promoting FoxP3^+^ Treg cell induction [11]. Interestingly, a number of studies have reported that B cells may have an active regulatory role in various parasitic infections. For example, IL-10-producing B cells in *L. major*-infected BALB/c mice are essential in suppressing type 1 responses that are necessary to clear infection [12] and *S. mansoni*-infected B cell-deficient μMT mice show more extensive hepatic granulomas [13]. In addition, in a model of systemic anaphylaxis or AAI, in combination with *S. mansoni* infection with adult stage worms only, B cells appeared to be major players in addition to IL-10 and Treg cells [7], [14].

In auto-immunity models several Breg subsets have been identified, including marginal zone (MZ), transitional or CD5^+^CD1d^hi^ B cells [15]. Recent studies in human auto-immune diseases have substantiated these findings by showing human Breg cells in peripheral blood characterized as CD24^hi^CD38^hi^ [16], CD24^hi^CD27^+^ [17] or CD1d^hi^ B cells [18].

Here, we investigated both in mice and humans whether schistosome infections can induce functional Breg cells. Indeed, we identified for the first time in peripheral blood of *S. haematobium*-infected children elevated numbers of IL-10-producing CD1d^hi^ regulatory B cells, which were decreased after treatment. The functional capacity of those schistosome-induced Breg cells was confirmed in a mouse model of allergic airway inflammation where Breg-derived IL-10 and Breg-induced Treg cells mediated suppression.

## Materials and Methods

### Ethics statement

Mice were housed under SPF conditions at the animal facilities of the Leiden University Medical Center in Leiden, the Netherlands. All animal studies were performed in accordance with the guidelines and protocols (DEC-07062, 07152, 08034, 09141) approved by the Ethics Committee for Animal Experimentation of the University of Leiden, The Netherlands. The human study was conducted according to the principles expressed in the Declaration of Helsinki. The study was approved by the “Comité d'Ethique Regional Independent de Lambaréné” (CERIL). Written informed consent was obtained from parents or legal guardians of children participating in the study.

### Animals

Six week-old female C57/Bl6 OlaHsd mice were purchased from Harlan. For the generation of chimeric IL-10-producing B cell-deficient mice, B cell-deficient μMT mice were irradiated to remove the bone marrow (BM). Subsequently, the irradiated mice were reconstituted with 80% of μMT BM and 20% IL-10^−/−^ BM cells (IL-10-deficient) or with 80% of μMT BM and 20% wild-type (WT) BM as described [10]. DEREG (DEpletion of REGulatory T cells) mice were kindly provided by Dr. T. Sparwasser [19].

### Parasitic infection and AAI induction

Mice were infected percutaneously with 40 *S. mansoni* cercariae and the infection lasted until 14 weeks (chronic phase starts around week 12)[8]. For AAI induction, mice were sensitized twice by i.p. injections of OVA (10 µg/mL, Worthington Biochemical Corp) in Imject Alum (2 mg/ml; Pierce) at 17 and 10 days before challenge. Sensitization was initiated during week 11 and 12 after the start of infection. Ten days after the last injection, mice received OVA aerosol challenges (10 mg/ml in PBS) for three consecutive days in the 14^th^ week of infection. Mice were sacrificed 24 hours after the last challenge. BAL fluids were collected and phenotyped by flow cytometry [8].

### Study population

Venous blood was obtained from 20 school children living in Lambaréné (Gabon) or from a nearby village (PK15) where *Schistosoma haematobium* is endemic. *S. haematobium* infection was detected by examining 10 ml of urine passed through a 12-µm-poresize filter (Millipore) and the eggs were stained with a ninhydrin solution. Children were classified as ‘infected’ if at least one *S. haematobium* egg was detected in the urine or ‘uninfected’ if three consecutive urine samples were negative. Infections with intestinal helminths *A. lumbricoides*, *T. trichiura* and hookworm were determined by analyzing one fresh stool sample using the Kato-Katz method [20]. Infection with *P. falciparum* was determined by PCR [21]. *S. haematobium*-infected children were treated with three doses of praziquantel (40 mg/kg) every two months. Intestinal helminth and malaria infected children received respectively a single dose of albendazole (400 mg) and an artemisinin-based combination therapy as per the local guidelines.

### Mouse cell purification and cell sorting

Single cell suspensions were prepared from the spleens, mesenteric lymph nodes, and mediastinal lymph nodes by dispersion through a 70-µm cell sieve (Becton Dickinson). Perfused lungs were minced to ∼1 mm pieces and digested by collagenase II/Dnase for 1 hour. The digested lungs were sequentially dispersed through 70- and 40-µm sieves. Erythrocytes were removed from the spleen and lung single cell suspensions by lysis. B cells were purified using anti-CD19 MicroBeads (Miltenyi Biotec). Follicular B cells (CD21^int^CD23^hi^) and marginal zone B cells (CD21^hi^CD23^int/low^) were stained with antibodies against CD19-PE (MB19-1, eBioscience), CD21-APC (7G6, BD Pharmingen), and CD23-FITC (B3B4, eBioscience) and separated using FACSAriaII cell sorting (Becton Dickinson). The sorted subsets were routinely ∼95% pure. For the depletion of MZ B cells, splenocytes were first incubated with CD19-PE (6D5, Miltenyi Biotec), followed by anti-PE multisort beads (Miltenyi Biotec) to isolate the B cells. Next, these beads were enzymatically removed and the isolated B cells were incubated with CD21-FITC (7G6, BD Pharmingen) antibody for 20 minutes. The CD21^hi^ MZ B cell fraction was depleted using anti-FITC magnetic beads (Miltenyi Biotec). For the total CD19^+^ B cells, the CD21^hi^ B cells were added back to the CD21^neg^ B cells (mock depletion). The depletion of MZ B cells was ∼92% pure. CD4^+^CD25^−^ T cells were enriched using anti-CD4 and anti-CD25 MicroBeads with a purity of 96% (Miltenyi Biotec).

### Human B cell isolation and characterization

PBMCs were isolated by Ficoll-Hypaque density gradient centrifugation from 20 ml of heparinized blood. B cells were isolated with anti-CD19 MicroBeads (Miltenyi Biotec) with a purity of ∼95%. For immunophenotyping different B cell subsets, isolated PBMCs were fixed in 2.4% PFA and stained for CD19-PB (HIB19, eBioscience), CD1d-PE (51.1, eBioscience), CD5-APC (UCHT2, BD), CD24-PeCy7 (ML5, ITK Diagnostics), CD27-APCeFluor780 (O323, eBioscience), and CD38-FITC (HIT2, BD).

### Adoptive transfer of isolated mouse B cells

Recipient mice were sensitized with two injections of OVA at day 0 and day 7. Ten days after the last injection, the OVA-sensitized animals received i.v. injection of 5×10^6^ pulmonary, mesenteric, splenic total or splenic CD19^+^ B cells depleted for MZ B cells from OVA sensitized-uninfected or OVA sensitized-infected mice. Blocking anti-IL-10R antibody (250 µg; kindly provided by Schering Plough Biopharma) or isotype control antibody was given i.p., one day before adoptive transfer. DEREG mice, which carry a Diphtheria toxin receptor-eGFP transgene under the control of an additional Foxp3 promoter, were treated with two diphtheria toxin (DT, 1 µg/ml) i.p. injections: one day before and two days after the adoptive transfer of B cells in order to deplete the FoxP3^+^ Treg cells. After two days, mice were challenged for three consecutive days and sacrificed 24 hours after the last challenge.

### *In vitro* mouse B cell stimulation and co-culture with CD4^+^CD25^−^ T cells

Mouse CD19^+^ B cells and B cell subsets (1×10^5^ cells) were cultured in the presence of SEA from *S. mansoni* eggs (20 µg/mL) for five days. Supernatants were stored for later cytokine analysis by ELISA. For *in vitro* Treg induction, B cells (1×10^5^ cells) were first irradiated with 2600 RAD and subsequently, co-cultured with CD4^+^CD25^−^ T cells (1×10^5^ cells) in the presence of medium or anti-CD3 (1 µg/ml) plus anti-CD28 (1 µg/ml). An isotype control anti-β-gal (10 µg/ml), or anti-IL-10 receptor (10 µg/ml) was added. After five days, cells were fixed according to the eBioscience FoxP3 fixation/permeabilization kit. Proliferation was confirmed by cell counts. Cytokines were measured in the cell culture supernatant using Luminex or ELISA (IL-1β,-4,-5,-6, -10, -12p40/70, -13, IFN-γ, TNF-α).

### Human B cell stimulation and intracellular staining for IL-10

Freshly isolated B cells (1×10^5^) were stimulated for 48 hours with 2.5 µg/ml anti-human IgG/IgM (Jackson ImmunoResearch) in the presence or absence of 10 µg/ml SEA from *S. haematobium* eggs. For ICS of IL-10, B cells were restimulated with PMA (50 ng/ml), ionomycin (2 µg/ml), and LPS (100 ng/ml; Invivogen) for 6 hours with the final 4 hours in the presence of BrefA (10 µg/ml; Sigma-Aldrich), followed by fixation with FoxP3 fixation/permeabilization kit and stained for CD1d-PE (51.1, eBioscience), CD20-APCeFluor780 (2H7, eBioscience), and IL-10-biotin (JES3-12G8, Abd Serotec) followed by second incubation with streptavidin-Qdot525 (Invitrogen).

### Statistical analysis

All murine results are expressed as mean ± SEM and the different groups were tested using the Student's *t*-test (two-tailed). Differences between infection groups in humans were tested by the Mann-Whitney U test. Differences within the same group pre- and post-treatment were compared by Wilcoxon matched pairs test. Probability values less than 0.05 were considered significant. *** p< 0.001, ** *p*<0.01, * *p*<0.05 and *# p*<0.1.

## Results

### IL-10-producing B cells are important for protection against allergic airway inflammation

**Figure 1:**
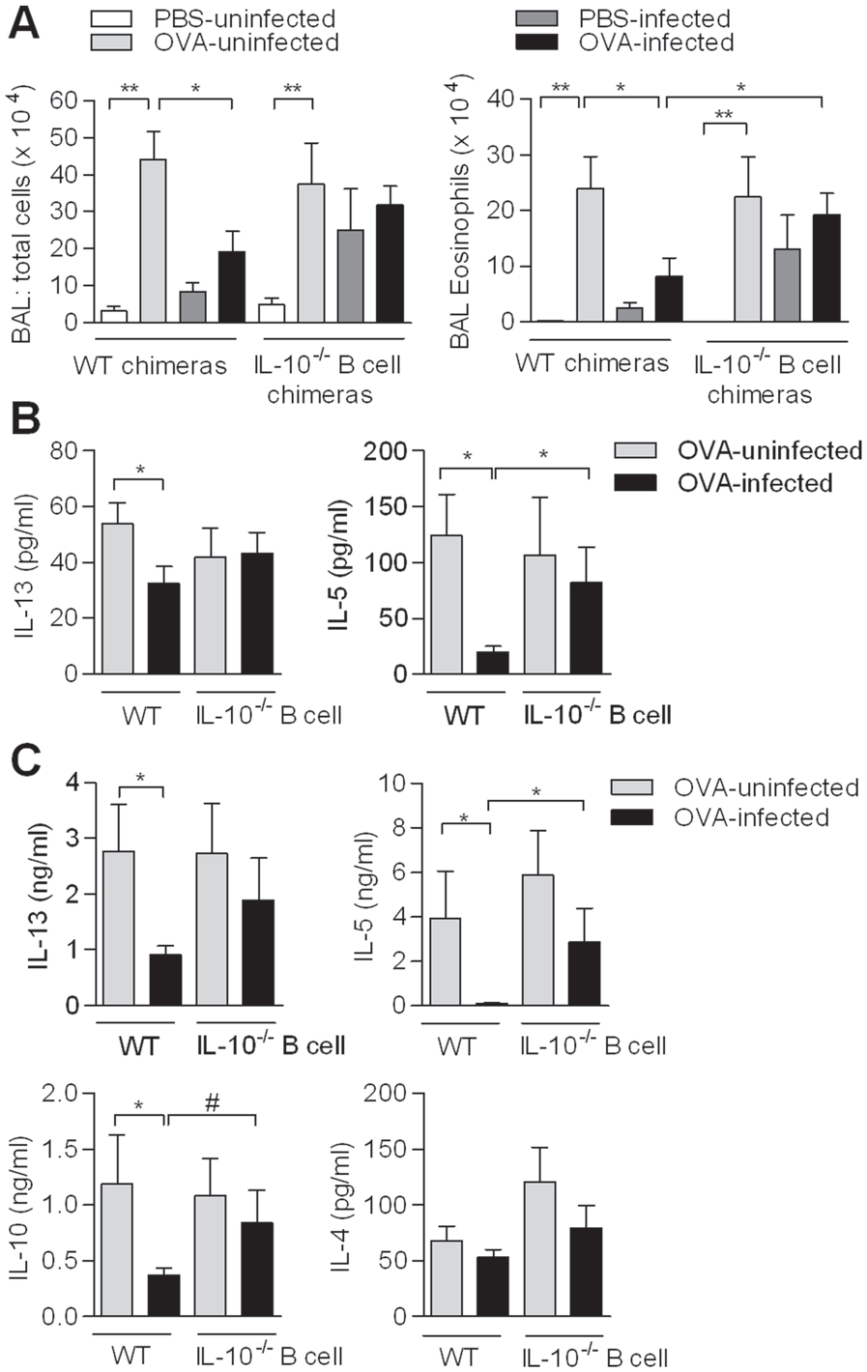
Role of IL-10-producing B cells on OVA-specific AAI during chronic *S. mansoni* infection. Chimeric WT and IL-10^−/−^ B cell mice were infected with *S. mansoni* and sensitized and challenged for OVA at week 11 and 12 after the start of infection. BAL fluid was collected and total BAL cells (A) and eosinophils were determined. (B) In the BAL fluid, IL-5 and IL-13 were measured by ELISA. (C) MedLN cells were collected and restimulated by OVA (10 µg/mL) for four days and IL-4, IL-5, IL-10, and, IL-13 production was determined using ELISA. Results are from two independent experiments and each group consists of 6 PBS- and 6 OVA-uninfected, 8 PBS- and OVA-infected mice.

Elevated IL-10 characterizes chronic stages of schistosome infection and is produced by B cells starting from week 12 during infection (Fig. S1)[8]. To determine whether B cells are a dominant source of IL-10 and whether this IL-10 is essential for protection against AAI during chronic schistosome infections, IL-10-deficient B cell and control wild-type (WT) chimeric mice were generated and chronically infected with *S. mansoni* followed by an allergic OVA sensitization and challenge. The IL-10-deficiency was restricted to the B cells population (confirmed by intracellular flowcytometry) as described before [10]. In the uninfected allergic groups (OVA-uninfected) for both WT and IL-10^−/−^ B cell chimeric mice, the bronchoalveolar lavage (BAL) fluid contained significantly more eosinophils (Fig. 1A), lymphocytes and macrophages compared to uninfected non-allergic groups (PBS-uninfected) (data not shown). Nevertheless, in OVA-infected WT mice significantly less eosinophils were found compared to OVA-uninfected WT mice (Fig. 1A), as previously described [8]. In contrast, eosinophilia in the OVA-infected group of the IL-10^−/−^ B cell mice was restored and was similar to the OVA-uninfected IL-10^−/−^ B cell group and significantly higher compared to the OVA-infected WT group. Interestingly, in the PBS-infected group, eosinophilia was equally high, suggesting that IL-10-producing B cells were involved in controlling non-allergic inflammatory processes during natural infections as well. Furthermore, IL-5, IL-13 and/or IL-10 were equally elevated in the BAL fluid and mediastinal lymph nodes (MedLN) of OVA-infected IL-10^−/−^ B cell mice compared to OVA-uninfected IL-10^−/−^ B cell group, whereas these Th2 cytokines were reduced in OVA-infected WT mice (Fig. 1B, C). The IL-5 production in the BAL fluid and by T cells in the MedLN was also significantly increased in the OVA-infected IL-10^−/−^ B cells mice compared to the OVA-infected WT mice as well. As expected, IL-4 remained at low levels due to the C57/Bl6 background of the chimeric mice (Fig. 1C). These results indicate that IL-10-producing B cells are critically involved in the downmodulation of eosinophilia and the Th2 response against OVA antigen leading to protection against AAI during chronic schistosomiasis.

### Pulmonary and splenic B cells produce IL-10 and protect against AAI

**Figure 2:**
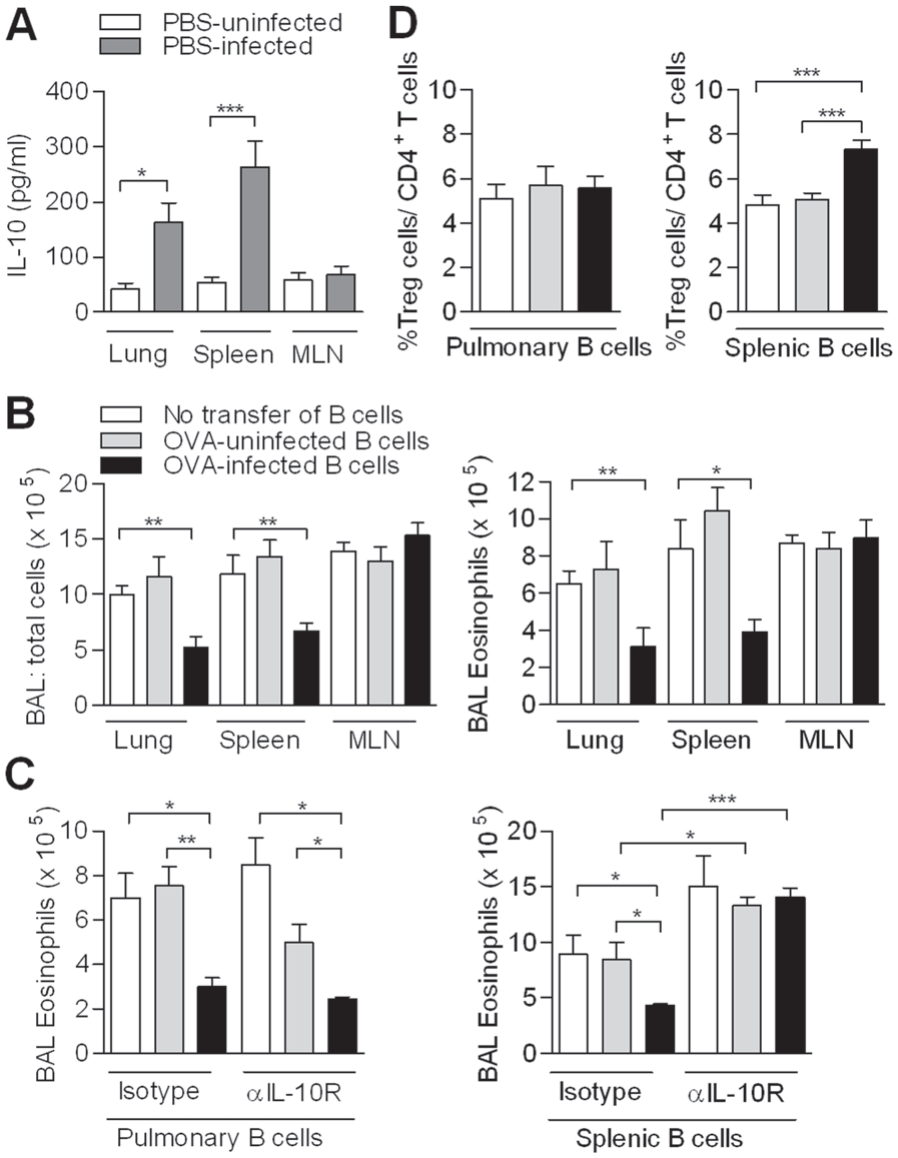
IL-10 production by B cells from different organs during chronic schistosomiasis and their role in protection against AAI. (A) WT mice were treated as in Fig. 1A. Splenic, pulmonary or mesenteric B cells (1×10^5^) were isolated and cultured in the presence of SEA (20 µg/ml) for five days. IL-10 production was measured using ELISA and medium value was subtracted. (B) OVA-sensitized recipient mice received 5×10^6^ B cells from different organs. After challenge, BAL cell numbers and eosinophils were determined (C) BAL numbers and eosinophilia of mice that received 250 µg isotype control or anti-IL-10R abs per mouse one day before the adoptive transfer. (D) The percentage of CD4^+^CD25^+^FoxP3^+^ Treg cells was determined in the lungs of recipient mice. Each graph represents three independent experiments, consisting of five mice per group.

In order to identify the dominant organ with IL-10-producing B cells during infection, we isolated B cells from organs that have previously been described to harbor regulatory B cells (spleen), drain schistosome infection sites (mesenteric lymph nodes, MLN) or are the effector site where allergic inflammation is found (lung). Both pulmonary and splenic B cells, but not mesenteric B cells, from chronically-infected mice were able to produce IL-10 upon soluble egg antigen (SEA) stimulation, with highest production by splenic B cells (Fig. 2A). To study the suppressive activity of isolated B cells from different organs in downmodulating AAI, we adoptively transferred CD19^+^ B cells from OVA-infected mice into OVA-sensitized recipient mice. AAI was reduced by pulmonary or splenic but not by mesenteric B cells (Fig. 2B). Interestingly, the protective effect of the transfer of splenic B cells, but not of pulmonary B cells, was abolished by administering a blocking IL-10 receptor antibody (Fig. 2C). Furthermore, we observed increased percentages of CD4^+^CD25^+^FoxP3^+^ T cells in the lungs of recipient mice, but only after administering splenic B cells (Fig. 2D). This data indicate that pulmonary B cells can drive IL-10 and Treg cell-independent protection against eosinophilic AAI, while splenic B cells protect via an IL-10-dependent mechanism and enhance local Treg cell numbers in the lungs.

### Splenic B cells induce Treg cells, which support reduction of AAI

**Figure 3:**
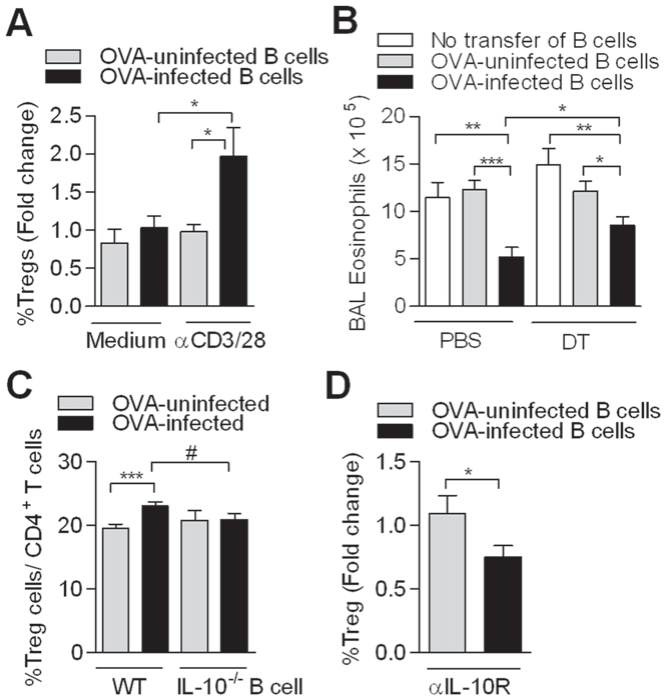
Treg cell induction by IL-10-producing Breg cells. (A) Irradiated splenic B cells (1×10^5^) were cultured with CD4^+^CD25^−^ T cells (1×10^5^) for 5 days in the presence of anti-CD3 and anti-CD28. Induction of CD4^+^CD25^+^FoxP3^+^ Treg cells (in %) by PBS-uninfected B cells was set at one. Fold change in Treg cell percentage for OVA-uninfected and OVA-infected B cells was calculated. Graph expresses results from three independent experiments. (B) OVA-sensitized DEREG mice were treated as in Fig. 2B in addition to a DT or PBS injection. This graph expresses two experiments, consisting of five mice per group. (C) WT and IL-10^−/−^ B cell chimeras were treated as in Fig. 1A. The MedLNs were collected and the percentage of CD4^+^CD25^+^FoxP3^+^ Treg cells was determined. Figure contains two independent experiments and each group consists of 6 to 8 mice. (D) *In vitro* co-culture were performed as described in (A) in the presence of blocking anti-IL-10R or isotype control antibodies. Percentage of Treg cells induced in the presence of isotype control was arbitrarily set at 1. Fold change in Treg cell induction in the presence of anti-IL-10R was calculated. Graph represents three independent experiments.

Regulatory B cells are known to regulate inflammation by recruitment and generation of Treg cells in auto-immune disorders [10]. Therefore, we cultured irradiated splenic B cells from OVA-uninfected or OVA-infected mice with CD4^+^CD25^−^ T cells from naive mice to evaluate Treg cell development. Splenic B cells from OVA-infected mice doubled the percentage of CD4^+^CD25^+^FoxP3^+^Treg cells (Fig. 3A), while pulmonary B cells did not change the level of Treg cells, as already confirmed *in vivo*. In addition, no shift towards Th1 or Th2 cytokines was found during the co-culture showing that the splenic B cells primarily influenced the development of Treg cells but not of other T cell subsets (data not shown).

Next, we studied the contribution of B cell-induced Treg cells in protection against AAI *in vivo* using OVA-sensitized FoxP3-DTR transgenic DEREG mice [19]. The temporal loss of FoxP3^+^ Treg cells during allergen challenge only partly restored AAI, showing that in the group with transferred B cells from OVA-infected mice, B cell-induced Treg cells are only partially involved in protection against AAI (Fig. 3B).

A causal relationship between IL-10-producing B cells and Treg cells has been suggested in antigen-induced arthritis model utilizing similar chimeric mice, where loss of IL-10-producing B cells led to significant reduction of Treg cells in draining inguinal LN [22]. In OVA-infected WT mice, we found more CD4^+^CD25^+^FoxP3^+^ Treg cells in the lung-draining MedLN compared to OVA-uninfected mice, which was not found in OVA-infected IL-10^−/−^ B cell mice (Fig. 3C). Using the same *in vitro* co-culture as describe above, significantly less Treg cell induction by splenic B cells was found when anti-IL-10R antibodies were added compared to isotype control (Fig. 3D), underlining the role of IL-10 for Treg cell induction both *in vivo* and *in vitro*.

### Schistosome-induced MZ B cells exhibit regulatory activities

**Figure 4:**
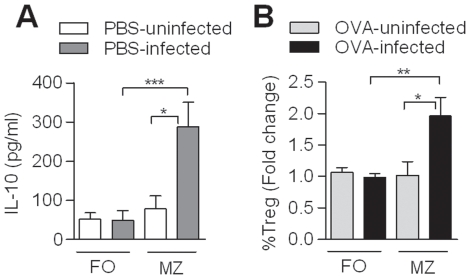
MZ B cells show regulatory features. (A) MZ and FO B cells from PBS-uninfected and PBS-infected were sorted using flow cytometry and cultured for 5 days in the presence of SEA for IL-10 production as in Fig. 2A. (B) Irradiated MZ B cells or FO B cells (1×10^5^) were co-cultured with CD4^+^CD25^−^ T cells as described in Fig. 3A. Treg cell induction by MZ or FO B cells from uninfected mice was set at one. Subsequently, fold change in Treg cell induction by MZ or FO B cells from OVA-uninfected and OVA-infected mice was calculated. Each graph contains three independent experiments with five mice per group.

We compared the two main splenic B cell subsets, the follicular (FO) B cells and the MZ B cells (which are high in CD1d, Fig. S2) for their ability to produce IL-10 during chronic infection. Sorted subsets from uninfected and infected mice were cultured in the presence of SEA, showing that the MZ B cells from infected mice produced high IL-10 levels, while FO B cells produced only low levels (Fig. 4A). Production of IL-12 and IL-1β remained low in both subsets of infected mice, while production of TNF-α and IL-6 was increased in FO cells, but remained low in MZ B cells from infected mice (Fig. S3). Based on our findings regarding Treg cell induction by total splenic B cells, we questioned whether MZ B cells, as the strongest IL-10 producers, were also responsible for Treg cell induction *in vitro*. Therefore, irradiated FO and MZ B cells were cultured with CD4^+^CD25^−^ T cells and the highest percentage of FoxP3^+^ T cells was observed in co-culture with MZ B cells (Fig. 4B), which was not explained by differences in proliferation as similar cell counts were found in cultures with either FO or MZ B cells (data not shown). These data indicate that MZ B cells are responsible for the regulatory features observed in total splenic B cells by producing IL-10 and by enhancement of FoxP3^+^ T cells.

**Figure 5:**
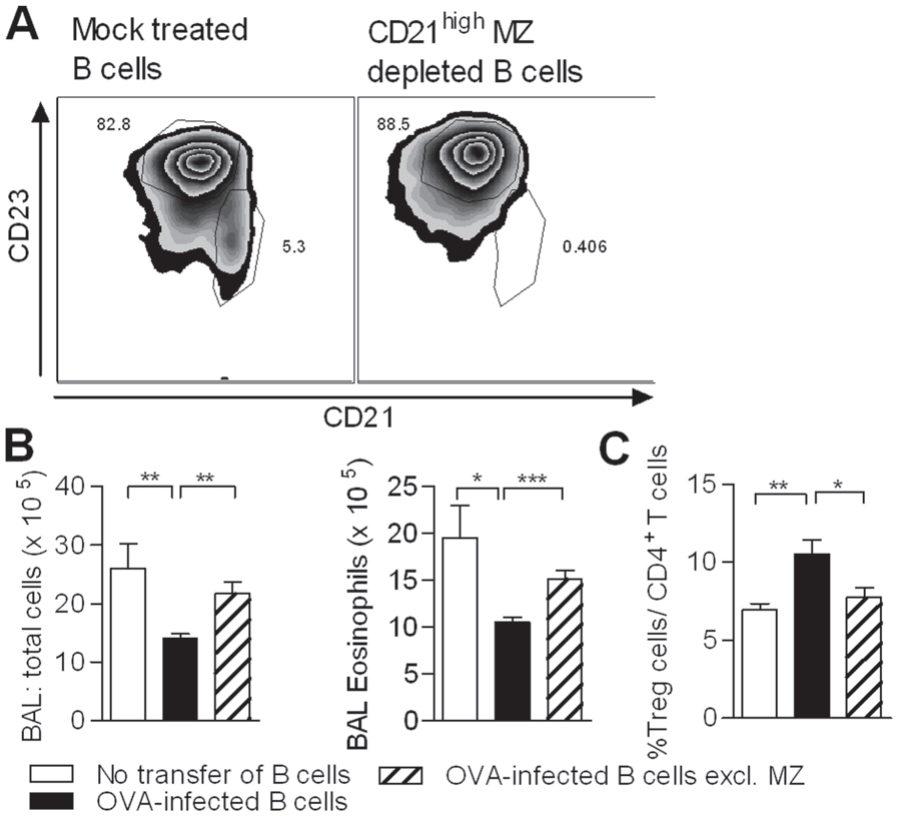
MZ B cells are important in the protection against AAI. (A) Mock-depleted splenic and the MZ-depleted B cells were injected in OVA-sensitized recipient mice. After challenge, total BAL cell count and the number of BAL eosinophils (B) and the percentage of CD4^+^CD25^+^FoxP3^+^ T cells in the lungs (C) was measured. Figure is representative of two independent experiments, consisting of five mice per group.

Next, the contribution of the MZ B cells *in vivo* was investigated by depletion of the CD21^hi^ cells from total splenic B cells of OVA-uninfected or OVA-infected mice by indirect magnetic labeling (Fig. 5A). The transfer of mock-treated B cells from OVA-infected mice resulted in a decrease in total BAL cell count and eosinophilia (Fig. 5B), as observed before. Importantly, the transfer of CD21^+^-depleted B cells restored the severity of AAI and the induction of pulmonary FoxP3^+^ Treg cells was lost (Fig. 5C), confirming the significance of MZ B cells in protection against AAI *in vivo*.

### Elevated levels of IL-10-producing CD1d^hi^ B cells in *S. haematobium*-infected Gabonese children

**Figure 6:**
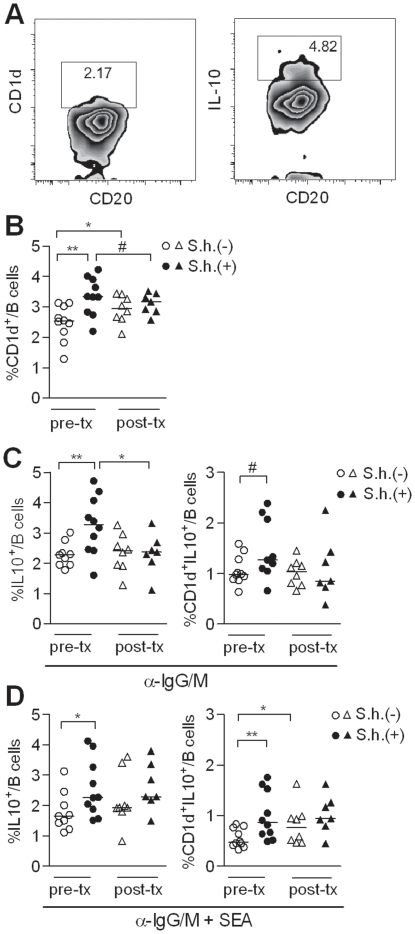
Presence of IL-10-producing CD1d^hi^ B cells during *S. haematobium* (S.h.) infection. (A) The CD1d^hi^ B cells and the IL-10 production of the total B cells were gated according to the gating strategy depicted in this graph. The gating of the IL-10 production of the different Breg subsets is similar as the total B cell gating. (B) PBMC were fixed and stained for Breg markers, including CD1d, and analyzed by flow cytometry. (C) Total peripheral blood B cells were cultured with anti-IgG/IgM or (D) anti-IgG/IgM plus SEA (10 µg/ml) for two days. Intracellular IL-10 production of the total B cells and the CD1d^+^ B cells was determined following PMA/Ionomycin/LPS and BrefA stimulation.

All data regarding schistosome-induced Breg cells stems from mouse models. Therefore, we asked whether in humans schistosomes can also induce IL-10-producing regulatory B cells. PBMC were collected from 20 Gabonese children that were either *S. haematobium* positive or negative (Table 1). All samples were simultaneously analyzed for different Breg markers, including CD24^hi^CD38^hi^, CD24^hi^CD27^+^ or CD1d^hi^(CD5^+^) (Fig. 6A). No differences were found between infected or uninfected donors for CD24^hi^CD38^hi^ or CD24^hi^CD27^+^ B cells (Fig. S4). However, significantly higher percentage of CD1d^hi^ B cells was found in infected children compared to uninfected children (Fig. 6B). There was also a trend for more CD1d^hi^CD5^+^ B cells in infected children, however, the total numbers were so low (<1%) that the reliability of this measurement can be questioned. Importantly, after six months of treatment with praziquantel, CD1d^hi^ percentages were reduced to levels comparable to the uninfected control group (Fig. 6B). Of note, CD1d^hi^ levels in the uninfected control group were significantly increased over the same period, which may reflect seasonal changes. Alternatively, 6 out of 8 donors from the uninfected group had an increased population of plasmablasts (CD19^+^CD24^lo^CD38^hi^ cells; Fig. S5), suggesting a recent, unrelated infection in Lambaréné, but not in the nearby village (∼15 km, PK15), which may have caused the increase in CD1d^hi^ B cells observed in the uninfected children.

**Table 1:** Demographic characteristics and infection status of Gabonese children.

	S.h.infected	S.h.uninfected
Participants pre-tx/post-tx	10/7	10/8
Mean age in years (range)	10.3 (8–14)	11.2 (8–14)
Sex male/female	5/5	4/6
Mean egg counts (range)	31.7 (1–201)	0
Co-infections:		
*Plasmodium falciparum*	4/10	1/10
*Ascaris lumbricoides*	3/9	3/8
*Trichuris trichiura*	3/9	6/8
Hookworm	1/9	1/8

Co-infections are depicted as number of participants infected out of total number of participants tested.

To compare the IL-10 production in B cells from infected and uninfected children, total peripheral blood B cells were stimulated with anti-IgG/IgM. Subsequent intracellular analysis showed an increase in IL-10-producing B cells in the total B cell population of infected children, which was significantly reduced after treatment (Fig. 6A,C). Further gating on the CD1d^hi^ B cell subset, confirmed that in particular CD1d^hi^ B cells from infected children produce more IL-10 as compared to uninfected children (Fig. 6C). Interestingly, we observed a slightly different pattern, when the cells were stimulated with anti-IgG/IgM plus SEA (Fig. 6D). Although also in this condition, both total and CD1d^hi^ B cells from infected children produce significantly more IL-10, this was not downregulated upon treatment. This may reflect the presence of a small, but persistent population of schistosome-specific B cells in the circulation of previously infected children that more readily produce IL-10 in response to SEA, as its cognate antigen, compared to the more aspecific stimulation with antibodies directed against IgG/IgM. Although it cannot be excluded that SEA may also act as an adjuvant, this seems to be less likely as only little IL-10 inducing activity of SEA is found in B cells from uninfected children. The fact that somewhat elevated IL-10 levels in anti-IgG/IgM plus SEA-stimulated B cells from uninfected group at post-treatment are observed compared to pre-treatment may again reflect seasonal effects or recent unrelated infections, suggested by the enhanced population of plasmablasts in this group (Fig. S5).

Altogether, these data confirm that also in humans, schistosomes can induce IL-10-producing Breg cells, of which the CD1d^hi^ B subset is the most prominent. Therefore schistosomes can be recognized as powerful Breg cell inducer in both mice and humans.

## Discussion

In this study we provide evidence that during chronic schistosomiasis IL-10-producing CD1d^hi^ B cells are induced in both humans and mice. In mice, schistosome-induced IL-10-producing Breg cells were necessary for protection against AAI as shown here in chimeric IL-10^−/−^ B cell mice. Splenic B cells were the most prominent source for IL-10. Interestingly, the induction of IL-10-producing splenic B cells by infectious agents has been previously documented for *L. major* [12], murine cytomegalovirus [23] and for *Schistosoma mansoni* by our group and by Amu *et al* [7]. These studies favor the concept that chronic infections, drive strong immunoregulatory processes in which IL-10-producing B cells seem to be important players (reviewed in [24]).

However, several reports have indicated that B cells can also suppress inflammation via IL-10-independent mechanisms. In a colitis model, mesenteric B cells were capable of reducing CD4^+^ T cell-dependent colon inflammation [25] and mesenteric CD23^hi^ B cells of *H. polygyrus*-infected mice were capable of inhibiting inflammation both in an EAE model and in an HDM-specific AAI model via an unknown mechanism [26]. Furthermore, studies in *S. mansoni*-infected μMT mice, where the lack of B cells led to increased liver pathology have suggested the involvement of FcR-dependent mechanisms [13]. Although their mode of action is still unknown, we describe here that transferred pulmonary B cells also protected against AAI independently of IL-10 and Treg cells. The putative role of antibodies or interaction with FcR has not been studied yet.

Further, we characterized the phenotype and function of the dominant IL-10-producing B cell subset, finding a regulatory function for CD1d^hi^ MZ B cells in the spleen of schistosome-infected mice. These data are in line with several other studies in auto-immunity models pointing towards MZ B cells as regulators of type 1 inflammation in SLE, ACAID or CHS [25], [27]. In addition, it was shown that CD1d^hi^-expressing splenic B cells reduced inflammation in a chronic colitis model [25] and AAI during ‘worm only’-schistosome infection [7]. However, within the spleen, other regulatory subtypes have been suggested; a rare CD1d^hi^CD5^+^ B cell subset, termed B10 cells, which are capable of downregulating inflammatory responses in a number of different auto-immune or contact hypersensitivity models [15] and transitional type 2 MZ precursor B cells (CD21^hi^CD23^hi^IgM^hi^), described in mouse models for CIA [22], [25] and SLE [28]. It is not fully clear whether these B cells are complete unique subsets because there is a substantial overlap between the (co-)expression of various markers, such as CD1d, CD5, CD21, CD23 and IgM. In addition, local inflammation or chronic infection may change the expression of individual markers complicating distinctions between the different proposed cell subsets.

Here we show that helminth-induced MZ B cells not only reduced allergic inflammation via IL-10 but also via the induction/recruitment of active FoxP3^+^ Treg cells. In humans, Treg cells are known to be an important element in reducing allergic inflammation as a mutation in the FOXP3 gene is associated with severe eczema, food allergy and high levels of IgE and eosinophilia [29]. Furthermore, children with asthma show quantitative and functional impairment of CD4^+^CD25^+^ Treg cells in the BAL fluid [30], while children that have outgrown their allergy have increased frequencies of allergen-responsive Treg cells [31], [32]. Therefore, having a system that embraces not only one regulatory system but in fact two seems to be a very efficient strategy to develop tolerance to environmental stimuli and prevent allergy. Indeed, in several studies Breg function has been linked to induction or recruitment of Treg cells, i.e. in a model of ACAID, colitis, EAE, SLE [24], [25] and allergic inflammation [7]. Importantly, in these studies including the one presented here, Breg-induced immune regulation does not fully depend on Treg cell activity, with the exception of the study by Amu *et al.* [7], where in fact an excess of regulatory CD1d^hi^ B cells was transferred to recipient mice on three consecutive days, in contrast to our study with only one B cell injection before the challenge.

Although the majority of studies on Breg cells have been conducted in mouse models there are now a few reports that confirm the existence of human Breg cells, in which both equivalents of already described ‘mouse’ Breg cells are identified in addition to some new subsets. Correale *et al.* have reported human IL-10-producing CD1d^hi^ B cells in helminth-infected MS patients [18]. However, these patients were infected with a mixture of different helminth species. Here, we now have established a causal relation between a single species of helminth, schistosomes and increased levels of IL-10-producing CD1d^hi^ B cells, which were reduced to ‘normal’ levels after anti-schistosome treatment. Of note, in both schistosome-infected and uninfected children, other geohelminths were found and for ethical reasons both groups were treated with the antihelmintic albendazole in addition to treatment with praziquantel for the schistosome-infected group. Despite their initial presence, we find reduced numbers of CD1d^hi^ B cells after treatment only in the schistosome-infected group but not in the schistosome-uninfected group. These data suggest that schistosomes have a more dominant effect on Breg cell induction than other gut-associated helminths. So far, two other human Breg subsets have been described, namely CD24^hi^CD27^+^ [17] and CD24^hi^CD38^hi^ Breg cells in healthy individuals, of which the activity of the latter was impaired in SLE patients [16]. Interestingly, we did not observe differences in these two Breg cell subsets in peripheral blood of infected versus uninfected children, indicating that schistosomes primarily induce CD1d^hi^ Breg cells. This discrepancy suggests that the various Breg cell subsets described so far may require different conditions for their development and activation.

As illustrated above, evidence from animal studies and a few human studies points towards a significant role for IL-10-producing Breg cells in modulating pathogenic hyperinflammatory responses. As such, it would be of great therapeutic interest if Breg cell activity could be specifically induced. For this, helminth infections may be of particular value, as *in vitro* exposure of splenic B cells to live schistosome worms or peritoneal injection of schistosome egg-derived glycans or filarial glycoproteins induces IL-10-producing B cells [7], [33]. However, the identification of the exact helminth-derived molecules involved, is a critical step as enhanced activity of Breg cells may form a valuable new target for therapy of rhinitis and/or allergic asthma.

## Supporting Information

Figure S1 **IL-10 production by CD19^+^ B cells during infection.** Mouse CD19^+^ B cells were isolated from the spleen at different time points during *Schistosoma mansoni* infection. The B cells were cultured in the presence of SEA from *S. mansoni* eggs (20 µg/mL) for five days. Supernatants were stored for IL-10 analysis by ELISA. This experiment represents one experiment with 3–4 mice per group.
(TIF)

Figure S2 **Geometric mean of CD1d fluorescence intensity on total B cells, FO and MZ B cells.**
(TIF)

Figure S3 **Production of cytokines after SEA stimulation.** MZ and FO B cells from PBS-uninfected and PBS-infected were sorted using flow cytometry and cultured for five days in the presence of SEA for IL-10 production as presented in Fig. 2A. In addition, we measured IL1-β, IL-12p40/70, IL-6 and TNF-α using Luminex.
(TIF)

Figure S4 **Percentage of CD24^hi^CD38^hi^. and CD24^hi^CD27^+^ B cells in peripheral blood of Gabonese children pre- and post-treatment, performed as described in legend to** **Fig. 6B** **.**
(TIF)

Figure S5 **Percentage plasmablasts in peripheral blood of Gabonese children pre- and post-treatment, performed as described in legend to** **Fig. 6B** **.**
(TIF)
