# Supplementary figures and images for: Schistosomes Induce Regulatory Features in Human and Mouse CD1dhi B Cells: Inhibition of Allergic Inflammation by IL-10 and Regulatory T Cells
Source: PLoS One. 2012 Feb 8;7(2):e30883. doi: 10.1371/journal.pone.0030883 (PMC3275567; doi:10.1371/journal.pone.0030883)

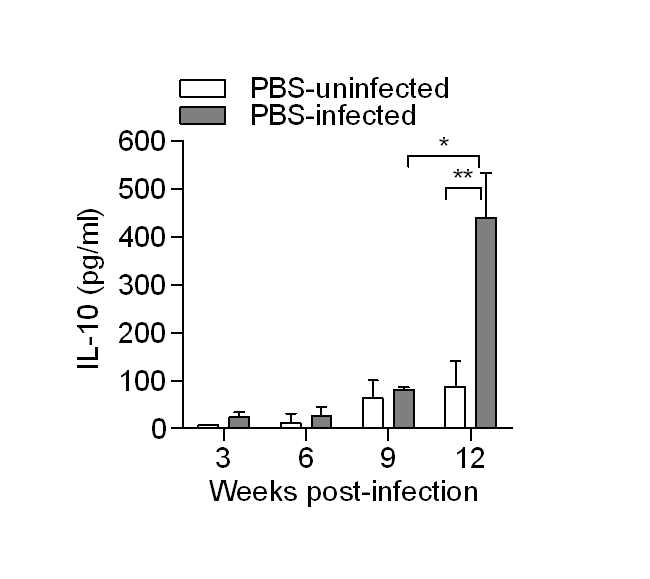

Supplement: Figure S1 — IL-10 production by CD19+ B cells during infection. Mouse CD19+ B cells were isolated from the spleen at different time points during Schistosoma mansoni infection. The B cells were cultured in the presence of SEA from S. mansoni eggs (20 µg/mL) for five days. Supernatants were stored for IL-10 analysis by ELISA. This experiment represents one experiment with 3–4 mice per group. (TIF) [file pone.0030883.s001.tif]

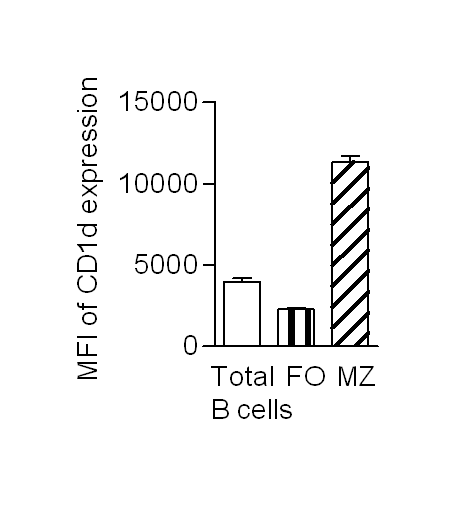

Supplement: Figure S2 — Geometric mean of CD1d fluorescence intensity on total B cells, FO and MZ B cells. (TIF) [file pone.0030883.s002.tif]

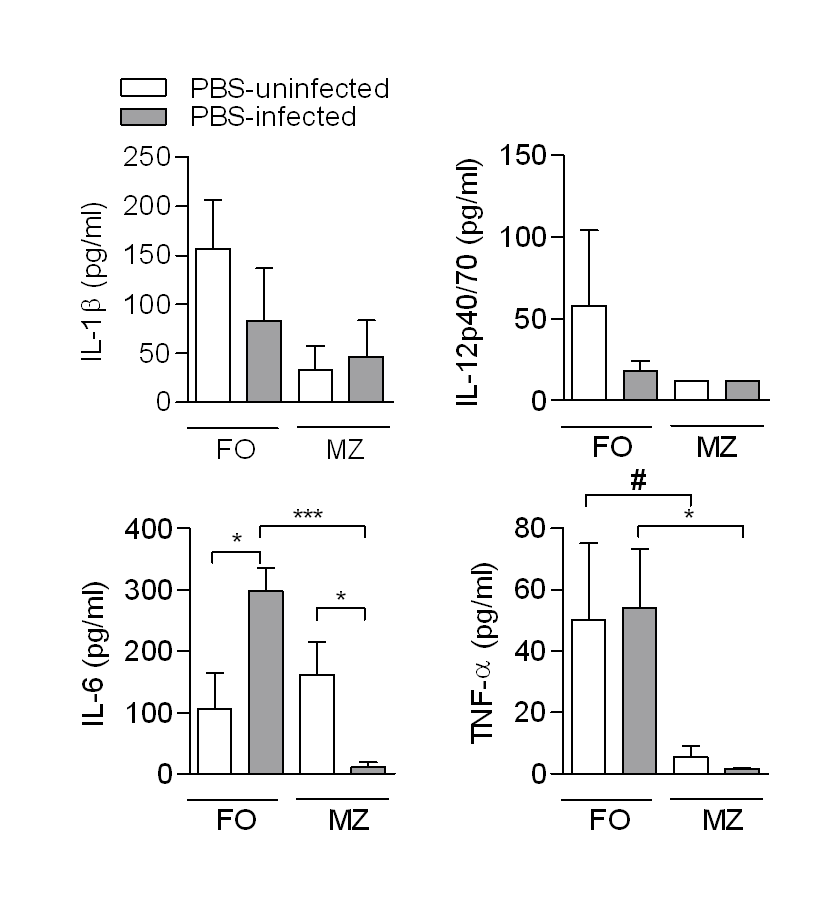

Supplement: Figure S3 — Production of cytokines after SEA stimulation. MZ and FO B cells from PBS-uninfected and PBS-infected were sorted using flow cytometry and cultured for five days in the presence of SEA for IL-10 production as presented in Fig. 2A. In addition, we measured IL1-β, IL-12p40/70, IL-6 and TNF-α using Luminex. (TIF) [file pone.0030883.s003.tif]

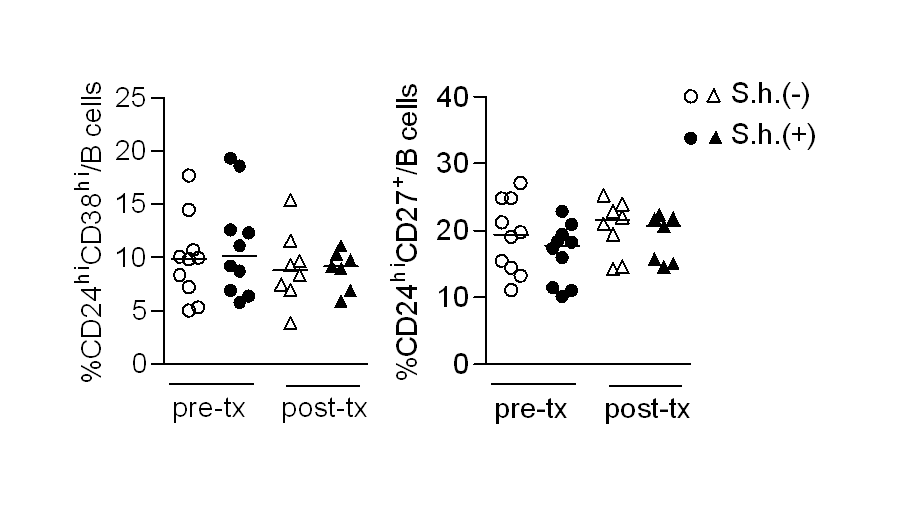

Supplement: Figure S4 — Percentage of CD24hiCD38hi. and CD24hiCD27+ B cells in peripheral blood of Gabonese children pre- and post-treatment, performed as described in legend to Fig. 6B . (TIF) [file pone.0030883.s004.tif]

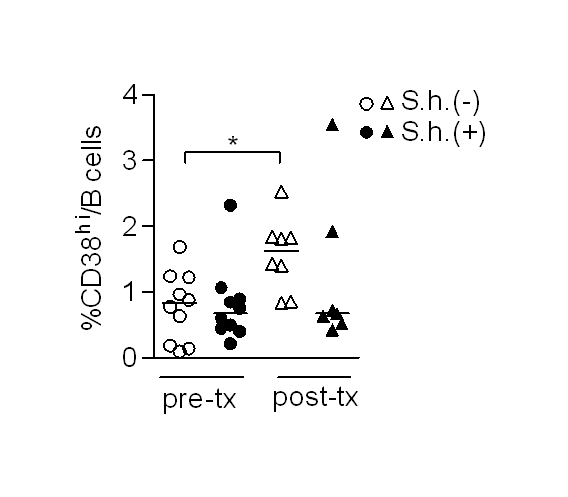

Supplement: Figure S5 — Percentage plasmablasts in peripheral blood of Gabonese children pre- and post-treatment, performed as described in legend to Fig. 6B . (TIF) [file pone.0030883.s005.tif]
